# A Contemporary Review of Barriers and Methods to Fostering Academic Urologists

**DOI:** 10.7759/cureus.50173

**Published:** 2023-12-08

**Authors:** Fumihiko Nakamura, Crystal Valadon, Zebulun Cope, Sriharsha Talluri, Brandon Chou, Jannah Thompson, Murali Ankem, Kellen Choi

**Affiliations:** 1 Department of Urology, University of Louisville School of Medicine, Louisville, USA; 2 Department of Obstetrics and Gynecology, University of Louisville School of Medicine, Louisville, USA; 3 Department of Urology, Michigan State University College of Human Medicine, Grand Rapids, USA

**Keywords:** barriers, aspiring, career, academia, education, medical, urologist, academic

## Abstract

Urology has shown a gradual decrease in the number of graduating residents who plan to pursue a career in academic medicine. Our objective was to identify barriers to academic urology, present options to mitigate those barriers, and explore strategic ways to encourage trainees to seek careers in academic urology. The authors performed a contemporary review of relevant articles through PubMed assessing prior survey studies, editorials, and expert opinion articles that evaluated academic urology, perceptions of academic medicine, physician burnout, and barriers that have been identified to pursuing careers in academic medicine. Selected articles were then independently reviewed by three authors for relevance and application of factors mitigating perceived barriers to pursuing a career in academic medicine, specifically academic urology. Barriers at the academic levels of medical school and residency were found to consist of the following: lack of exposure to research early in their medical careers, inadequate mentorship, all-specialty leading levels of burnout, current average levels of medical school indebtedness contrasted to perceptions of pay disparity when compared to private practice urologists’ income, and perceptions of difficulty in maintaining the academic “triple threat.” More acutely, the decision to make Step 1 a pass/fail exam, with the addition of historically low match rates in urology, have resulted in additional complications and concerns for aspiring academic urologists. There are clear barriers that graduating urology residents encounter when considering a career in academic medicine. In this review, we present possible mitigating factors that may be instituted at the individual, medical school, and postgraduate levels to increase the number of practicing academics.

## Introduction and background

The gradual decrease in the number of clinician-educators throughout the academic community over the years has been a discouraging and persistent trend [1]. Urology is not immune to the effects of this shift. According to the American Urological Association (AUA) 2018 resident census, only 35% of urology residents intended to pursue a career in academic medicine [2]. This low recruitment rate could prove problematic for the academic urology community in future years as departments are already experiencing the negative impacts of academic physician shortages. This is evident by the fact that 53.5% of chairs and division heads have been searching for two or more years to fill available positions [3]. Recruiting and retaining a lower percentage of trainees within the academic realm negatively impacts research endeavors, clinical training experiences, and the future growth of the urological field as the multitude of emerging medical technologies are largely devised, implemented, and promoted by physicians in academic medicine.

Haas et al. provided an in-depth evaluation of the many obstacles that may dissuade potential academicians from joining academia [4]. The goal of this contemporary update is to identify and expand our understanding of these barriers in the context of impactful changes facing entry into urology today. Identifying the sources of these difficulties is crucial to understanding the importance of implementing effective response strategies to prevent the probable loss of academic physicians in the future.

## Review

Challenges

Medical School

The mindset of pursuing an academic career can develop during the earliest stage of medical training: medical school. Therefore, it is prudent for the medical education community to implement strategies that expose medical students to opportunities that teach them more about a career in academic medicine.

Current medical school curricula in many institutions may limit students’ ability to gain adequate research experience representative of a career in academic medicine through their coursework. Several studies have shown that students who participate in research activities during medical school are more likely to be interested in pursuing a career in academia. Influence from an academic mentor or role model was also shown to have a positive impact on a student’s consideration of academic medicine; however, access to such an individual may not always be readily available [5]. Having access to mentorship creates an invaluable experience for medical students to explore academic medicine as a career path.

Another factor to consider is medical student indebtedness. Greysen et al. showed that although there has not been a substantial change in the proportion of students graduating with debt since 1985, the mean level of indebtedness has increased significantly to $158,000 per student in 2011 [6]. As a comparison, the average medical indebtedness in 1984 was $26,500 [7]. This value has only continued to increase over recent years. According to the Association of American Medical Colleges (AAMC), the mean educational debt for the Class of 2021 was $203,062, which does not include any student loan debt students might have taken on before medical school [8]. To accurately understand how much medical student indebtedness has increased, inflation must be accounted for. Therefore, after adjusting the 2021 average debt to “1984 dollars,” the value of $203,062 is equivalent to $79,103.37 in 1984 [7]. Higher levels of debt have been shown to be a deterrent for physicians who may choose to pursue a career in academic medicine. Careers in academic medicine have a reputation for lower financial compensation compared to private practice settings [9].

Residency

Although some medical students may have had the opportunity to participate in a significant amount of research, a large number of students are not exposed to any meaningful research until they enter residency. However, even after matriculating into residency, research experiences may vastly differ depending on the curriculum structure of the individual program. While research activities are required for Accreditation Council for Graduate Medical Education-accredited programs, the amount of time dedicated to research responsibilities varies drastically among urology residencies. This may range from a complete absence of dedicated research time to a full year of protected time to pursue research goals. Those programs with little to no dedicated time require residents to perform research on their own time in addition to busy clinical responsibilities. This dichotomy also impacts the scale and rigor of research projects that can be accomplished. The type of program residents attend can influence their opinion regarding academic medicine and potentially place them on a trajectory toward a career that aligns with their individual urology program’s strengths instead of the individual’s strengths.

A resident’s decision to pursue fellowship training may also be an indicator of interest in academia; however, it may be difficult to initially discern whether that interest in academia is inherent to personal desires, or simply to improve their chances of matching into a fellowship [10]. Regardless, a fellowship typically allows more time to pursue research endeavors as the likelihood of having dedicated research time and a research mentor increases. Therefore, these individuals may have a greater tendency to become an academic physician. A survey conducted among urology residents found that there were three main independent factors positively associated with choosing a fellowship: participating in a shorter residency program (five vs. six-year program), successfully publishing a manuscript during residency, and receiving mentorship [11]. Lin et al. sought to identify perceived obstacles to a career in academic medicine as it pertained to trainees at the resident level. Their results indicated that a lack of mentorship was the most significant barrier reported by residents, followed by concerns about work/life balance, job availability, lack of autonomy, and competing roles [12].

Physician Parents in Academia

Physicians who are parents must seek to find an adequate balance between managing their work and home responsibilities effectively. One aspect of this balance is identifying adequate childcare arrangements for their children. In the case of physicians, weekly work hours generally average more than 40 hours, and on-call responsibilities can demand more than childcare services that operate at “usual hours” can offer. Oftentimes, ensuring that their children are adequately looked after may interfere directly with their careers. In fact, having to accommodate childcare responsibilities at the expense of work schedules has been reported in up to 87% of women and 59% of men in dual-physician marriages [13]. As a result of their additional parental responsibilities, physician parents may not be able to fulfill the concept of the ideal worker, an individual who works long hours and shows devotion to their work by putting it ahead of all other aspects of their life, that is often idealized in the workplace [14]. A substantial and directly competing choice must often be made regarding the time spent parenting versus the time spent performing and advancing one’s medical career. In the case of academic medicine where a practicing urologist has not only clinical and surgical responsibilities but also research requirements that exceed most physicians in private practice, the increased work-hour demands stand in contrast to a desirable work-life balance and time to parent face-to-face.

Triple Threat Concept

The triple threat concept denotes the ability to successfully manage being an effective clinician, educator, and investigator [15]. Historically, in academia, a physician had to achieve a high degree of functioning in all three arms to be considered for promotions in sought-after positions. While this tactic was often employed in the 1960s among physicians practicing, it began to markedly lose favor as the work demands of the ideal “triple threat” were found to result in a decrease in both job and personal satisfaction among academic clinicians. Ultimately, the stress associated with maintaining the triple threat status led to the gradual avoidance of academia throughout the medical community [16].

Difficulty in obtaining the triple threat can be attributed to the increasing complexity of modern medicine. The pace of quickly emerging clinical advances requires devotion of a significant amount of time to stay up to date on the most recent and relevant aspects of patient care. Excellent medical educators must invest a substantial amount of effort in acquiring knowledge and developing skills to be effective teachers. Increased competition among research funding has led to principal investigators using at least 80% of their overall work hours to produce a successful research project [15]. Additionally, administrative duties such as billing and paperwork that have increasingly been thrust upon physicians add a fourth barrier to the idea of the “triple threat” [17].

Measures to be taken

Medical School

A key factor to consider is the declining match rate in urology. From 2019 to 2022, the match rate has steadily dropped from 84.8% to 65.6% [18]. Though it is generally understood that match rates across all competitive specialties undergo a natural pattern of fluctuation, the timing of a historically low match year coinciding with the turbulence of significant changes in medical education warrants further study.

To cultivate interest in academic medicine among medical students, programs should seek to provide ample research exposure, research opportunities, and mentorship. An effective method that can be adopted by medical schools is to generate a platform that allows students to easily contact and interact with willing academic physicians within their local community. However, simply providing access to academic clinicians only serves those who are already considering this career path and is unlikely to be beneficial in drawing potential students. In addition to networking databases, medical schools could incorporate talks on academic medicine into their initial student orientations, emphasizing its crucial importance and getting students to think about their various options from the start. Miller et al. also suggested that creating either a formal or informal mentorship program would provide improved guidance, leading to higher-quality research experiences among medical students [19]. Promoting these programs to individuals during the formative stages of their careers would provide them with the opportunity to generate productive and meaningful professional relationships within the academic realm. These relationships and connections can be invaluable along the trajectory of a physician’s career.

Regarding the substantial debt that medical students accumulate, the American Medical Association and AAMC continue to make efforts to improve their financial burden by seeking the expansion of loan repayment programs [20]. Loan repayment programs exist in a variety of types (scholarships, forgiveness, and repayment) and are offered through both state and national programs. For example, a loan repayment award of up to $50,000 per year is offered by the National Institute on Minority Health and Health Disparities to awardees who conduct nonfederal research about health disparities for a minimum of two years [21]. Another option available to physicians is the public service loan forgiveness program that forgives the remaining balance on a Direct Loan after 120 payments have been made while working full time for a qualified employer, who is either a government or not-for-profit organization [22]. A portion of the options offered can be accessed directly via the AAMC website; however, students should discuss available options with the financial aid advisor at their institution to receive a more comprehensive list.

Additionally, medical schools and residencies should include within their educational agendas some discussions on financial matters as they pertain to physicians. Quite often, the discussion of physician finances, including expected physician income, debt management strategies, additional income opportunities, and home/family planning, as it pertains to personal finances, is perceived as a taboo subject within medical education. Physicians are notoriously unprepared for a realistic financial trajectory, which includes an understanding of their debt within the framework of their “take home” income [23]. A fear of academic medicine is that it not only includes a lower income compared to private practice but also requires additional years of training while continuing to receive a resident-level salary. Financial education and/or planning may provide concrete numbers that assuage fears of inadequate income in academic medicine. At its best, medical schools and/or residencies should consider providing one-on-one financial planning that takes into account career, family, and investment goals.

Residency

The barrier most commonly stated by residents influencing them to avoid pursuing academic medicine was a lack of adequate mentorship [11,12]. This could be interpreted as a lack of appropriate guidance relative to research and balancing the work/life ratio in academia. Research is a tedious process that may be difficult to bring to completion as there are many obstacles to traverse during the process, including developing an innovative idea, successfully navigating the institutional review board system, undergoing multiple revisions to suit various publication requirements, and so forth. Residents, by nature of the clinical demands of training, are already limited in the time they can devote to other pursuits. A mentor can help navigate the arduous learning curve of a research project and help the resident avoid some of the common pitfalls of research. This should help new researchers avoid investing a significant amount of time without comparable returns.

Identifying mentors within the program who are willing to devote time and resources to cultivating a productive research environment is crucial. Students should be proactive when applying to residency in seeking programs with strong mentor-mentee relationships, and likewise, residency programs should put forth efforts to cultivate and incentivize those relationships. Successful mentors will properly guide residents through their research path but will also instill the knowledge and confidence necessary to complete the process independently. Rustgi et al. proposed including the following components on that pathway to promote success: monthly meetings with intentional action plans after each meeting; instructions on manuscript writing, PowerPoint presentations, and grant writing; introduction to regulatory affairs; and promoting an effective work-life balance [24].

An academic mentor who is able to give individualized attention and work closely with a resident illuminates the positive aspects of choosing a career in academia and serves as a consistent motivator to achieve their goals. Mentors will also benefit from these interactions. It has been shown that it can improve the mentor’s level of career satisfaction [25]. Overall, mentorship can positively impact faculty retention rates [26] and remove the greatest barrier that opposes residents pursuing a career in academia.

Physician Burnout

Despite burnout being well documented throughout medicine, particularly in the field of urology, instrumental changes have yet to be made throughout the majority of programs and practices. A study conducted by Chouhan et al. surveyed 464 urologists regarding burnout and only 33 (7.1%) stated that their workplace had specific interventions in place to address this growing concern [27].

Resources such as resilience training, support programs, structured mentorship programs, and access to mental health services have been associated with a lower rate of burnout [28-30]. Promoting wellness in general is a low-cost change that institutions can implement to positively impact the lives of urologists [30]. Surgeons, in general, still have a “grit and grind” mentality, and a few presentations on burnout, while instructional, meet the necessary requirements for properly developing skills to combat burnout.

More emphasis should be placed on successfully developing coping mechanisms. Namely, improvement stems from active membership and constant communication regarding skill development and the creation of lifelong habits. Most urology programs are small, so individualized attention is quite feasible. Personalized resiliency training for every resident based on their likes and dislikes would help instill stronger habits to live a healthy and balanced life.

A key component to reducing the risk of burnout is the understanding of control. A recent study revealed that clinicians who grasped and focused on life elements that were within their control while accepting those elements out of their control were less likely to experience burnout [29]. In other words, selectively “choosing your battles” to ones that can be influenced and/or changed by the physician had a positive correlation to avoiding burnout [11]. Other changes such as working as a team, seeking advice, and reading a non-medical book once per month have also been shown to mitigate the occurrence of burnout [28-30].

Assuredly, urology as an entire specialty should seek to take action toward mitigating burnout within the field. This initiative has the potential to retain more clinicians, recruit future urologists, and directly combat the expected shortage of 3,880 urologists by 2030 which is currently projected [31,32].

Physician Parents in Academia

Options that provide career flexibility with a more desirable work-life balance present as attractive options to recruited candidates. An example of work options that are currently implemented throughout the medical community include but are not limited to the following: availability of part-time work, alternative work schedules, leave for family care and childbearing, and extensions regarding the timing for tenure. The presence of such policies has proven to be key in recruiting and retaining physicians within academic medicine. Therefore, academic institutions should examine the expansion of the traditional work week and model [14]. The widespread presence within academic institutions of easily accessible childcare that adapts to the long and often unpredictable physician work hours would dissuade worries about finding adequate childcare in the pursuit of career betterment and/or fulfillment. Physician parents during residency often struggle with arriving on time to a closing childcare facility or scrambling to find adequate childcare. Residents who are not parents witness the frustrations of their colleagues, and this may deter them from academia if they assume these frustrations will be the norm. An academic institution with the foresight to provide childcare commensurate with the career hours of a physician shows a willingness to partner with a physician toward individual career fulfillment [13]. The modern era requires consideration of the working parent along with acknowledgment that both men and women are now more focused on achieving a desirable work-life balance.

Triple Threat Concept

Although the flaws of the triple threat concept have been exposed, many within the academic community feel the pressure to strive toward this ideal. Currently, the robust breadth of information and expectations within each arm of the triple threat presents an unrealistic aim in achieving not only competency but also excellence in each of the three domains. Instead, the focus should be shifted from viewing this as a goal to be achieved by a single individual to a divisional one. The well-known adage “divide and conquer” is certainly apropos in this matter. Encourage members within the department to pursue areas of interest that they are both passionate about and best suited for [16]. Implement communication strategies to facilitate an effective exchange of information allowing individuals who possess expert knowledge in each arm to benefit from one another [33]. Transforming the pursuit of the triple threat to a divisional goal presents a reflection of the current wealth of information in medicine and a representation of the evolution of the acquisition and dissemination of this vast knowledge.

## Conclusions

The obstacles that challenge the future recruitment of urologists into academic medicine are not insurmountable. Identifying and implementing methods of improvement that are targeted at overcoming current barriers is paramount to preserving this endangered field. Fostering healthy work environments, providing adequate resources, offering research experience, placing emphasis on quality mentorship, and striving toward increased career flexibility are several tactics that can be employed. Initiating these changes reflects the need and desire to re-envision an ever-progressing and evolving construct of academia. Our best, brightest, and most passionate should witness the joy of academic medicine in their journey to contribute to the specialty’s growth of knowledge and application. We have the ability to remove barriers, inspire careers, and contribute to the achievement of career goals while reducing burnout. Regardless of what career path urologic residents ultimately choose, striving to provide a postgraduate educational experience with those aims is worth pursuing.
